# Knowledge and practice on adequate sunlight exposure of infants among mothers attending EPI unit of Aleta Wondo Health Center, SNNPR, Ethiopia

**DOI:** 10.1186/s13104-019-4221-4

**Published:** 2019-03-29

**Authors:** Asres Bedaso, Melese Gebrie, Bedilu Deribe, Mohammed Ayalew, Bereket Duko

**Affiliations:** College of Medicine and Health Sciences, School of Nursing, Hawassa University, Hawassa, Ethiopia

**Keywords:** Sunlight exposure, Mothers’ knowledge, Mothers practice, Vitamin D

## Abstract

**Objective:**

The main objective of this study was to assess knowledge and practice of adequate sunlight exposure of infants among mothers attending EPI unit at Aleta Wondo Health Center, Sidama zone, SNNPR, Ethiopia. Institutional based descriptive cross sectional study design was used. 313 mothers who had under 1 year child and immunization follow-up were selected by simple random sampling technique using immunization registration book as sampling frame.

**Result:**

Out of 313 respondents identified for the study 98.03% (n = 307) were responded for the interview. From the total respondents 279 (90.9%) of respondents exposed their infants to sunlight but only 62 (22%) of them exposed adequately. From 307 mothers, 191 (62.2%) are knowledgeable about sunlight exposure and 91 (32.6%) of mothers had good practice of exposing their infants to sunlight.

**Electronic supplementary material:**

The online version of this article (10.1186/s13104-019-4221-4) contains supplementary material, which is available to authorized users.

## Introduction

Sunlight exposure has a lots of health benefits for infants, it helps the body to produce vitamin D that helps the body to absorb calcium [1]. Also has a function of strengthening bones thereby preventing rickets in children and osteomalacia in adults and possibly inhibiting growth of some cancers [2]. Visible sunlight to the eyes gives health benefits through its association with timing of melatonin synthesis; maintenance of normal and robust cardiac rhythms and reduce the risk of seasonal affective disorder [3]. A daily requirement of vitamin D can be obtained by 30–60 min exposure to sunlight in the morning [4].

It is important to limit the sun exposure between the hours of 10 a.m. and 4 p.m. for infants. In infants 1 week after birth the level of vitamin D is related to the level in their mothers during pregnancy [5]. The other source of vitamin D is from ingested diet and from supplements [6]. Adequate supplementation of vitamin D prevents low birth weight (LBW), birth asphyxia and deafness due to premature birth [7]. Studies worldwide identify lack of sun exposure as the main cause of rickets [8].

Rickets is a major public health problem in many countries of the world and it is common in children in Sub-Saharan African countries. Infants at risk of rickets are those whose mothers had poor vitamin D status during pregnancy and those exclusively breast-fed for a prolonged period with little skin exposure to ultraviolet B (UVB) [9]. Rickets is usually manifests as skeletal abnormalities, including frontal bossing, craniotabes, widening wrist, bowed legs and rachitic rosary [10]. The major causes of nutritional rickets in Ethiopia is lack of exposure to sunshine and inadequate intake of vitamin D [11].

Inadequate sunlight exposure of infants combined with nutritional rickets continues to be an evolving problem with several causes [12]. Nutritional rickets has received considerable attention from public health specialists in a number of developed countries. In developing countries, attention has been focused on rickets because of its effect on bone growth and mineral homeostasis and because of its association with increased infant and childhood mortality especially when accompanying lower respiratory tract infections [13, 14].

Primary deficiency is highly prevalent, even in countries with abundant sunshine, when skin exposure to UVB sunlight is limited by lifestyle and other factors. In Ethiopia, a review of rickets stated that the prevalence of rickets was highly as 40%, making it one of the highest in the world [15]. A study conducted in Kenyatta National Hospital showed that 58.8% of children aged 6 months develop rickets [16]. A recent study conducted in Kiambu District Hospital states that the prevalence of rickets in children 0–59 months to be 3.4% [17].

Until recently, little attention has been paid to the prevalence of rickets in most countries, but it is clear that rickets has been and remains a problem in Northern Asian countries, Middle East and in a number of countries in Africa [18]. A study done in Debre Markos 93% of mothers exposed their infants to sunlight but only 57.9% of them exposed daily [19]. A review of pediatric admissions in Jimma hospital, south western Ethiopia indicated that about 10% of children were diagnosed has rickets [20].

Mothers’ plays a key role in prevention of rickets by exposing their infants to sunlight adequately. Therefore, the objective of this study was to assess the knowledge and practice of adequate sunlight exposure of infants among mothers attending EPI unit at Aleta Wondo Health Center, Sidama zone, SNNPR, Ethiopia and fill the gap in these areas of concern.

## Main text

### Methods

#### Study design, area and period

An institutional based descriptive cross sectional study design was employed. Aleta Wondo is a town which is found in Sidama zone, SNNPR, Ethiopia. The town is located about 345 km away from Addis Ababa and 64 km from the regional capital city, Hawassa. This study was conducted from February 1 to April 30, 2018.

#### Source population and study participants

All mothers who attend EPI unit of Aleta Wondo health center for under one children EPI service were source population. All mothers who attend EPI unit of Aleta Wondo health center for under one children EPI service during the study period and fulfill the inclusion criterion were considered as study population.

#### Sample size determination and sampling technique

The sample size was calculated using a single population proportion formula by estimated prevalence of 54% taken from the study conducted in Debre Markos Town [19], with 5% marginal error (d) and confidence interval of 95% (Z α/2 = 1.96). Based on these assumptions and adding 10% non-response rate, the total estimated sample size was 312.

Regarding sampling, first, we selected one health center among all health centers through simple random sampling technique. Finally, simple random sampling technique was used to select the required number of participants among those who fulfill the inclusion criteria.

#### Inclusion and exclusion criteria

Mothers who attend EPI unit of Aleta Wondo health center for under one children EPI service were included in the study. Mothers who have difficulty of communication (hearing problem) were excluded from the study.

#### Data collection

Structured and pre-tested questionnaires were used to collect data. The questionnaire had four parts such as socio demographic related question, knowledge related question, practice related question and other determinant factors. The data was collected by trained nurses.

#### Data analysis

Data was entered and statistical analysis was carried out using SPSS version 20. The collected data were presented by frequency and percentage using tables, bar graph and pie charts. Mean and standard deviation was computed for numerical variables.

#### Operational definitions

Adequate sunlight exposure: Those mothers who exposed their infants to sunshine at morning 8–10 a.m. for 15–30 min. Knowledgeable: those mothers who scored more than the mean value of response for knowledge related questions. Good Practice: Those mothers who scored more than the mean value of response for practice related questions.

### Result

#### Socio demographic characteristics

All the required 313 study participants who came for EPI service at Aleta Wendo health center immunization clinic were interviewed with the response rate 100%. 144 (46.2%) of the respondents were between the ages of 22–27 years and 256 (82.1%) of their infants age were below 6 months. The mean age of the mothers and infants were 24 years and 4 months respectively (Table 1).

**Table 1:** Socio-demographic characteristics of mothers who attend EPI service in Aleta Wendo Health Center, Aleta Wondo Town, Southern Ethiopia, 2018 (N = 312)

Variable	Category	Number	Percent (%)
Mothers age	16–21	119	38.1
	22–27	144	46.2
	28–33	43	13.8
	34 +	6	1.9
Infant’s age	0–6 months	256	82.1
	7–12 months	56	17.9
Religion	Protestant	205	65.7
	Orthodox	65	20.8
	Muslim	36	11.5
	Other	6	2.0
Ethnicity	Sidama	233	74.7
	Oromo	33	10.6
	Amhara	36	11.5
	Other	10	3.2
Marital status	Single	5	1.6
	Married	298	95.5
	Divorced	4	1.3
	Widowed	5	1.6
Mothers educational status	Unable to read and write	26	8.3
	Able to read and write	11	3.5
	Grade 1–6	55	17.6
	Grade 7–10	147	47.1
	Grade 11–12	17	5.4
	Diploma and above	56	17.9
Family size	1–3	146	46.8
	4–6	130	41.7
	≥ 6	36	11.5
Mothers occupation	Student	12	3.8
	Housewife	216	69.2
	Government employee	49	15.7
	Private employee	8	2.6
	Daily laborer	5	1.6
	Merchant	22	7.1
Husbands educational status	Unable to read and write	22	7.1
	Able to read and write	14	4.5
	Grade 1–6	27	8.6
	Grade 7–10	139	44.6
	Grade 11–12	26	8.3
	Diploma and above	84	26.9
Childhood history of common cold	Yes	167	53.5
	No	145	46.5
Childhood history of pneumonia	Yes	60	19.2
	No	252	80.8

#### Maternal information about the need of sunlight exposure of infants

**Fig. 1:**
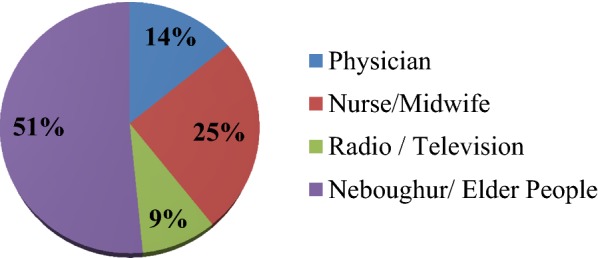
Maternal source of information about the need of sunlight exposure of infants among who attend EPI service in Aleta Wendo Health Center, Aleta Wondo Town, Sidama zone, SNNPR, Ethiopia, 2018 (n = 288)

Among the total respondents, majority 288 (92.3%) of the mothers had information about the need of sunlight exposure for their infants and out of those mother who had information, half 148 (51.0%) of mothers got the information from neighbors/elders (Fig. 1).

#### Maternal knowledge about sunlight exposure of infants

From total respondents, 265 (84.9%) of mothers reported that sunlight exposure had positive benefit for infants and from those mothers. Majority, 157 (59.3%) of women’s indicated that sunlight exposure was useful to strength bone. In the other side, nearly half 152 (48.7%) of the study subjects indicated that sunlight exposure had negative or harmful effect on infant (Table 2).

**Table 2:** Knowledge status of mothers about sunlight exposure of their infants among who attend EPI service in Aleta Wendo Health Center, Aleta Wondo Town, Southern Ethiopia, 2018 (N = 312)

Variable	Categories	Number	Percent (%)
Maternal positive belief towards the benefit of sunlight exposure of infants	Yes	265	84.9
	No	47	15.1
Benefit of sunlight exposure (n = 265)	Strengthen bone	157	59.3
	Strengthen teeth	18	6.8
	Strengthen body	18	6.8
	Produce vitamin D	56	21.1
	Keep child warm	16	6.0
Maternal perception harmfulness of sunlight exposure of infants	Yes	152	48.7
	No	160	51.3
Harmful effects of sunlight exposure (N = 152)	Skin cancer	67	21.5
	Sun burn	30	9.6
	Inflammation and Hyper pigmentation	21	6.7
	Blindness	26	8.3
	Sterility	8	2.6
Good time to expose infants to sunlight	Morning time	282	90.4
	After noon Time	15	4.8
	Evening time	8	2.6
	Any time	7	2.2
Awareness about the effect of inadequate or absence of sunlight exposure such as rickets	Yes	204	65.4
	No	108	34.6
Awareness attribute to detection of rickets (sign of rickets) (N = 204)	Late closure of fontanel	56	27.5
	Late tooth eruption	18	8.8
	Bowing of leg	63	30.9
	Enlargements of joints	24	11.8
	Knock knee	30	14.7
	Weak leg	9	4.4
	Don’t know	4	2.0
Knowledge attribute to prevention and/or curability of rickets (N = 204)	Yes	156	76.4
	No	35	17.2
	Don’t know	13	6.4
Treatment or prevention option of rickets (N = 204)	Follow doctors advice	107	52.5
	Early detection and treatment	19	9.3
	Adequate Diet Intake	25	12.2
	Sunbathing the child	30	14.7
	Don’t know	23	11.3

#### Mothers general knowledge about adequate sunlight exposure of infants

The mean score value of mothers who had knowledge about sunlight exposure was 6 out of the total score 10. More than half 196 (62.8%) of the study participants were knowledgeable about sunlight exposure (Additional file 1).

#### Practice of mothers about sunlight exposure of infants

Out of the total respondents, 250 (80.1%) of mothers exposed their infants to sunlight. Among the reasons for not exposing their infants to sunlight, 62 (19.9%) of mothers said it is due to fear of cold and 19 (30.7%) responded due to fear of evil eyes (Additional file 2).

#### Age of infants for first sunlight exposure

From all mothers who exposed their infants to sunlight, 75 (30%) of mothers started exposing their infant to sun light at age of 45 days and above (Additional file 3).

#### Mothers general practice level about adequate sunlight exposure of infants

The mean score value of mothers who had practice of sunlight exposure of infants was 5 of the total score 10. More than half 145 (58%) of the study participants had good practice about adequate sunlight exposure (Additional file 4).

### Discussion

In the current study, more than half (62.8%) of the mothers were knowledgeable about of sunlight exposure for infants. In addition most of mothers (84.9%) had awareness of the positive health benefit of sunlight exposure for infant and majority of them (59.3%) indicated sunlight exposure as the most important source of vitamin D used for growth and strength of bone through mobilization of calcium in the body. Also, 65.4% of the mother had awareness about the effect of inadequate or absence of sunlight exposure in health status that leads to rickets. This finding was consistent with the study done in Turkey which indicated that exposure of infant for sunlight was beneficial for bone development, diaper rash and neonatal jaundice [21].

Concerning the health risk of solar UV radiation exposure, nearly half (48.7%) of mothers believed that sunlight exposure had negative health effect as a result of improper time and duration of exposure, such as skin cancer and sun burn. This idea was also supported with a review of research done in Germany on the challenges resulting from positive and negative effects of sunlight [22]. This indicated that most of the mothers do not have enough information about healthy sun bathing and its effect since sun bathing might be also useful for treating neonatal jaundice, dipper rash and prevention of vitamin D deficiency related conditions like hypertensions, cardiovascular disease and bone disease etc [23].

Regarding practice of sunlight exposure, 58.0% of the study participants had good practice about adequate sunlight exposure who score more than or equal to mean score value. However, majority (80.1%) of mothers were exposed their infants to sunlight. This finding was lower than similar study done in Debre Markos Town of Ethiopia which was 93% [19]. The reason for the difference might be the difference in socio cultural factors like fear of illness, fear of evil eyes and witchcraft and level of appropriate information concerning sunning the baby.

In this study, only 17.6% of mothers were started sun light exposure of their infants between 0–15 days of neonatal life, which was lower than similar study done in Debre Markos town of Ethiopia which was 24% [19]. However, 67.6% of mothers of this study were sunning their babies at daily which was better than the study done at Debre Markos town of Ethiopia which was 57.9% [19]. In addition, more than half (63.6%) of the mothers exposed their babies in outdoor which were lower than the similar study done previously at Debre Markos town which was 89.4% [19].

Most of mothers (90.0%) were exposed their babies between the time range of 8–10 a.m. in this study, but 54.8% and 26.8% of mothers were sunning their babies without sun screening or clothing and with time duration of 15–30 min as recommended, respectively. This finding was slightly lower than the study done at Debre Markos on exposing without sun screening and time of exposure which accounts 57.3% and 99.1%, respectively; whereas, its higher on duration of exposure which accounts 25.5% [19]. This study finding was also incomparable with similar study done in Dale woreda of Southern Ethiopia as shown that 41.1% of the mothers exposed their child to sunlight within 1 month of birth [24].

Most of the children were exposed to sunlight for 20 to 30 min per day. About 49.4% were exposed to sunlight for 7 days in a week and 90.0% children were exposed to sunlight without clothing.

In this study, most of the mothers (90.8%) were used lubricants to be applied on their infant’s body when exposed to sunlight. Beside this, 41.4% of mothers were applied lubricants during the time of sunlight exposure, and followed by mothers who were applied lubricant after sunlight exposure which accounts 38.7%. This finding was lower than the same study done at Debre Markos town as shown that most (98.4%) of respondents applied lubricants on the baby’s body at the time of sunlight exposure and majority (70.6%) of mothers apply during sunlight exposure [19].

### Conclusion

Although majority of the respondents have good knowledge about importance of sunlight exposure for the infants but there is a gap on time to start sunlight exposure and time to stay on sunlight while exposing.

## Limitation of the study

Since the study is cross sectional it does not show cause and effect relationship between dependent and independent variables.

## Additional files

**Additional file 1.** Distribution of maternal general level of knowledge about sunlight exposure of their infants based of knowledge score among who attend EPI service in Aleta Wendo Health Center, Aleta Wondo Town, Southern Ethiopia , 2018 (n = 312).

**Additional file 2.** Practice of mothers on adequate sunlight exposure of their infants among who attend EPI service in Aleta Wendo Health Center, Aleta Wondo Town, Southern Ethiopia, 2018 (N = 250).

**Additional file 3.** Age of infants who sunlight exposure among mothers who attend EPI service in Aleta Wondo Health Center, Aleta Wondo Town, Southern Ethiopia , 2018 (N = 250).

**Additional file 4** General Practice of mothers among who expose their infants to sunlight and who attend EPI service in Aleta Wendo Health Center, Aleta Wondo Town, Sidama Zone, Southern Ethiopia, 2018 (n = 250).

## Abbreviations

CI: confidence interval
EPI: expanded program immunization
LBW: low birth weight
SNNPR: Southern Nations Nationality and Peoples Region
SPSS: Statistical Package for Social Science
UV: ultra violet
