# Supplementary material for: Knowledge and practice on adequate sunlight exposure of infants among mothers attending EPI unit of Aleta Wondo Health Center, SNNPR, Ethiopia
Source: BMC Res Notes. 2019 Mar 29;12:183. doi: 10.1186/s13104-019-4221-4 (PMC6440125; doi:10.1186/s13104-019-4221-4)
Supplement: Supplementary file 1 — Additional file 1. Distribution of maternal general level of knowledge about sunlight exposure of their infants based of knowledge score among who attend EPI service in Aleta Wendo Health Center, Aleta Wondo Town, Southern Ethiopia , 2018 (n = 312). [file 13104_2019_4221_MOESM1_ESM.docx]

Additional file 1: Distribution of maternal general level of knowledge about sunlight exposure of their infants based of knowledge score among who attend EPI service in Aleta Wendo Health Center, Aleta Wondo Town, Southern Ethiopia , 2018 (n=312)
