# Supplementary material for: Knowledge and practice on adequate sunlight exposure of infants among mothers attending EPI unit of Aleta Wondo Health Center, SNNPR, Ethiopia
Source: BMC Res Notes. 2019 Mar 29;12:183. doi: 10.1186/s13104-019-4221-4 (PMC6440125; doi:10.1186/s13104-019-4221-4)
Supplement: Supplementary file 2 — Additional file 2. Practice of mothers on adequate sunlight exposure of their infants among who attend EPI service in Aleta Wendo Health Center, Aleta Wondo Town, Southern Ethiopia, 2018 (N = 250). [file 13104_2019_4221_MOESM2_ESM.docx]

**Additional file 2:** Practice of mothers on adequate sunlight exposure of their infants among who attend EPI service in Aleta Wendo Health Center, Aleta Wondo Town, Southern Ethiopia, 2018 (N=250)

| **Variables** | **Categories** | **Frequencies** | **Percent (%)** |
| --- | --- | --- | --- |
| **Do you expose your baby on sunlight**  **(N=312)** | Yes | 250 | 80.1 |
|  | No | 62 | 19.9 |
| **Factors for not to expose infants to sunlight (N=62)** | Fear of witchcraft | 15 | 24.2 |
|  | Fear of evil eye | 16 | 25.8 |
|  | Sickness of the child | 7 | 11.3 |
|  | Fear of cold | 19 | 30.7 |
|  | Fear of Pneumonia | 5 | 8.1 |
| **Frequency of exposure** | Daily | 169 | 67.6 |
|  | Sometimes | 81 | 32.4 |
| **Place where expose the baby to sunlight** | Indoor | 91 | 36.4 |
|  | Out door | 159 | 63.6 |
| **Time of the day exposing infants to sunlight** | Morning 8-10am | 225 | 90.0 |
|  | Midday 11am-1pm | 21 | 8.4 |
|  | Afternoon 2-4 pm | 4 | 1.6 |
| **Condition of clothing during exposure** | Unclothed | 137 | 54.8 |
|  | Completely covered | 42 | 16.8 |
|  | Partially covered | 45 | 18.0 |
|  | With diaper and eye protection only | 26 | 10.4 |
| **Length of time of exposing infants to sunlight(Amount of time spent in the sun)** | 5-10 min | 80 | 32.0 |
|  | 10-15 min | 86 | 34.4 |
|  | 15-30 min | 67 | 26.8 |
|  | Above 30 min | 17 | 6.8 |
| **Application of lubricants on the body during sunlight exposure** | Yes | 227 | 90.8 |
|  | No | 23 | 9.2 |
| **Time of lubricant application (N=227)** | Before exposing | 45 | 19.8 |
|  | During exposing | 94 | 41.4 |
|  | After exposing | 88 | 38.7 |
| **Types of lubricant used (N=227)** | Vaseline | 165 | 72.7 |
|  | Baby lotion | 42 | 18.5 |
|  | Butter | 16 | 7.0 |
|  | Other oil | 4 | 1.8 |
|  |  |  |  |
