# Supplementary material for: Knowledge and practice on adequate sunlight exposure of infants among mothers attending EPI unit of Aleta Wondo Health Center, SNNPR, Ethiopia
Source: BMC Res Notes. 2019 Mar 29;12:183. doi: 10.1186/s13104-019-4221-4 (PMC6440125; doi:10.1186/s13104-019-4221-4)
Supplement: Supplementary file 3 — Additional file 3. Age of infants who sunlight exposure among mothers who attend EPI service in Aleta Wondo Health Center, Aleta Wondo Town, Southern Ethiopia , 2018 (N = 250). [file 13104_2019_4221_MOESM3_ESM.docx]

Additional file 3: Age of infants who sunlight exposure among mothers who attend EPI service in Aleta Wondo Health Center, Aleta Wondo Town, Southern Ethiopia , 2018 (N=250)
