# Supplementary material for: Knowledge and practice on adequate sunlight exposure of infants among mothers attending EPI unit of Aleta Wondo Health Center, SNNPR, Ethiopia
Source: BMC Res Notes. 2019 Mar 29;12:183. doi: 10.1186/s13104-019-4221-4 (PMC6440125; doi:10.1186/s13104-019-4221-4)
Supplement: Supplementary file 4 — Additional file 4 General Practice of mothers among who expose their infants to sunlight and who attend EPI service in Aleta Wendo Health Center, Aleta Wondo Town, Sidama Zone, Southern Ethiopia, 2018 (n = 250). [file 13104_2019_4221_MOESM4_ESM.docx]

Additional file 4: General Practice of mothers among who expose their infants to sunlight and who attend EPI service in Aleta Wendo Health Center, Aleta Wondo Town, Sidama Zone, Southern Ethiopia , 2018 (n=250).
